# Odor Recognition Memory in Parkinson's Disease: A Systematic Review

**DOI:** 10.3389/fnagi.2021.625171

**Published:** 2021-05-25

**Authors:** Tom Eek, Maria Larsson, Nil Dizdar

**Affiliations:** ^1^Department of Neurology in Linköping, and Department of Biomedical and Clinical Sciences, Linköping University, Linköping, Sweden; ^2^Gösta Ekman Laboratory, Department of Psychology, Stockholm University, Stockholm, Sweden

**Keywords:** odor recognition memory, Parkinson's disease, dementia, olfaction, systematic review

## Abstract

Olfactory impairment is a central non-motor symptom in Parkinson's disease (PD). Previous studies have demonstrated that olfactory dysfunction is associated with mental illness and impaired cognition. The frequently investigated olfactory functions are odor detection, discrimination, and identification. However, few studies have focused on odor recognition memory (ORM). ORM tasks involves episodic memory which therefore can facilitate the detection of dementia among patients with PD and consequently adjust their treatment. Thus, the aim of this systematic review is to summarize the existing research on ORM in PD. Databases and reference lists were used for data collection. Studies were included in the review if they met the eligibility criteria derived from the PICOS-framework. Quality evaluation of the studies was based on the STROBE-statement. Six studies with small samples were included in the analysis which demonstrated the scarce research on the subject. The studies targeting ORM were heterogenous and involved two main tasks: odor recognition and odor matching. The synthesis of the data demonstrated that PD patients performed significantly lower than controls on both tasks, especially on odor matching task. Only the odor recognition task exhibited a difference between patients with PD vs. Alzheimer's disease (AD). PD patients performed significantly better than AD patients. The findings based on the available limited data support the notion that odor recognition task can be of importance in identifying Parkinson's disease dementia (PDD). To investigate this hypothesis, future research needs to include larger samples of PD, PDD and AD patients executing the same odor recognition task.

## Introduction

Parkinson's disease (PD) is primarily identified by the evident debilitating motor symptoms: tremor, bradykinesia, rigidity, and postural instability. In 2015 ~6 million people were reported to suffer from the disease worldwide, a number estimated to increase rapidly to 12 million by 2040 due to the increased elderly population (Dorsey et al., 2018). Nonetheless, often non-motor symptoms (NMS) as depression, cognitive decline, sleep disorder, constipation, and olfactory dysfunction are present years before diagnosis and have been significantly associated with poor quality of life (Chaudhuri et al., 2006). The prevalence of the olfactory dysfunction in PD is estimated to be 50–90% and therefore one of the most studied NMS of the disease (Fullard et al., 2017). In fact, according to Braak's theory the parkinsonian neurodegeneration begins in the olfactory bulb and spreads toward other main parts of the cerebral olfactory system (Doty, 2012). For example, patients with PD show an extensive alpha-synuclein pathology in anterior olfactory nucleus (AON), cortical nucleus of the amygdala, piriform cortex, olfactory tubercle, and entorhinal cortex. Another possible explanation for this dysfunction is the distorted levels of typically affected neurotransmitters in PD such as dopamine, acetylcholine, and serotonin (Fullard et al., 2017).

Against this background, testing olfactory function may aid identifying PD in its premotor phase and thus allowing assessment of innovative treatments that requires early detection such as stem-cell therapy. The sense of smell is frequently assessed with psychophysical tests. One of the most well-established and comprehensive smell tests is the “Sniffin Sticks” which includes measurements of *odor detection, odor identification*, and *odor discrimination* (Hummel et al., 2007). A later development of the “Sniffin Sticks” is the Test of Odor Memory (TOM) which aims to measure *episodic odor recognition* (Croy et al., 2015). Prior studies targeting these olfactory functions showed variation between different tasks regarding brain activity, performance among PD patients and sensitivity to the parkinsonian progression (Fullard et al., 2017). Hence, different tasks can aid in decision making regarding various clinical questions. Additionally, the variation in performance between the tasks indicate that each task put different demands on olfaction, cognition, and memory (Hedner et al., 2010).

The odor detection task aims to measure the participant's olfactory sensitivity. Therefore, it is primarily based on a bottom-up processing and assumed to be a prerequisite for higher odor functions (Boesveldt et al., 2009). Conversely, the odor identification task which requires from the participant to verbally label common odors, is associated with higher cognitive function such as semantic memory. This type of memory refers to the participant's knowledge of facts about the world. The odor discrimination task involves also higher cognitive functions, especially working memory. It requires from the participant to create olfactory representations, storage them in short-term memory and compare between the different representations (Larsson, 2002; Hedner et al., 2010).

In the episodic odor recognition task, a set of odors (olfactory targets) that the participant is asked to memorize are presented at the encoding phase. At the retrieval phase the targets are presented again intermixed with new odors (olfactory distractors). The participant is required to determine if the presented odor is a distractor or a target. The research on odor recognition is remarkably limited, nevertheless the hypothesized dominant cognitive function in this task is episodic memory (Croy et al., 2015). Episodic memory is a top-down process and refers to the intentional retrieval of information, which is associated with temporal and spatial information (Larsson, 2002).

In summary, this review focuses on odor recognition memory (ORM) in PD due to multiple reasons. Previous studies did not demonstrate a correlation between other odor tasks and classical episodic memory tasks, while the type of cognitive load in the ORM task has still not been adequately examined (Hedner et al., 2010). The integrity of episodic odor memory may be particularly sensitive for pathological disturbances as dementia (Larsson, 2002). The risk of developing cognitive impairment and dementia is high in PD, especially in the advanced phase of the disease. Previous studies showed that dementia eventually will occur in up to 83% of PD patients (Hely et al., 2008). Furthermore, some antiparkinsonian treatments (e.g., dopamine agonists and deep brain stimulation in subthalamic nucleus) have been associated with deterioration of cognitive function, making the importance of detecting *de novo* dementia more crucial (Witt et al., 2013). The ORM task has also been associated with a wide cerebral activity and therefore is preferable regarding detection of dementia in a multisystem disease such as PD, minimizing the risk of missing initial cognitive symptoms (Savic et al., 2000). Although the ORM task may play a crucial role in early detection of PDD and consequently influence the treatment of the disease, the research on ORM in the context of PD is remarkably limited. Therefore, the aim of this review is to summarize the existing research on ORM in PD and generate research questions for future studies.

## Methods

### Literature Search

The Preferred Reporting Items for Systematic Reviews (PRISMA) guided the literature search (Moher et al., 2009). To identify relevant records from 1975 and onwards, three databases: Web of Science, Medline, and PsycINFO were used from January–May 2020. Studies were also collected from other sources (e.g., studies reference lists). Since Medical Subject Headings (MeSH) which describes accurately the topic of the review had not been found, well-established and broad terms in the research field were investigated. This strategy permitted a more inclusive search and therefore suitable for an unstudied subject. The terms *odor memory* OR *odor recognition* were used in AND-combination with *Parkinson's disease* OR *Parkinsons disease* OR *PD*.

### Inclusion and Selection Procedure

The eligibility criteria were derived from the parameters incorporated in the PICOS-framework: population, intervention, comparison, outcome, and study type (Moher et al., 2009). Studies were included if they met the following criteria:

(1) Included human subjects with PD.
(2) Explicitly intended to measure odor memory.
(3) Compared either between different olfactory functions, populations, or both.
(4) Reported results on odor memory.
(5) Designed as an empirical study.

**Figure 1:**
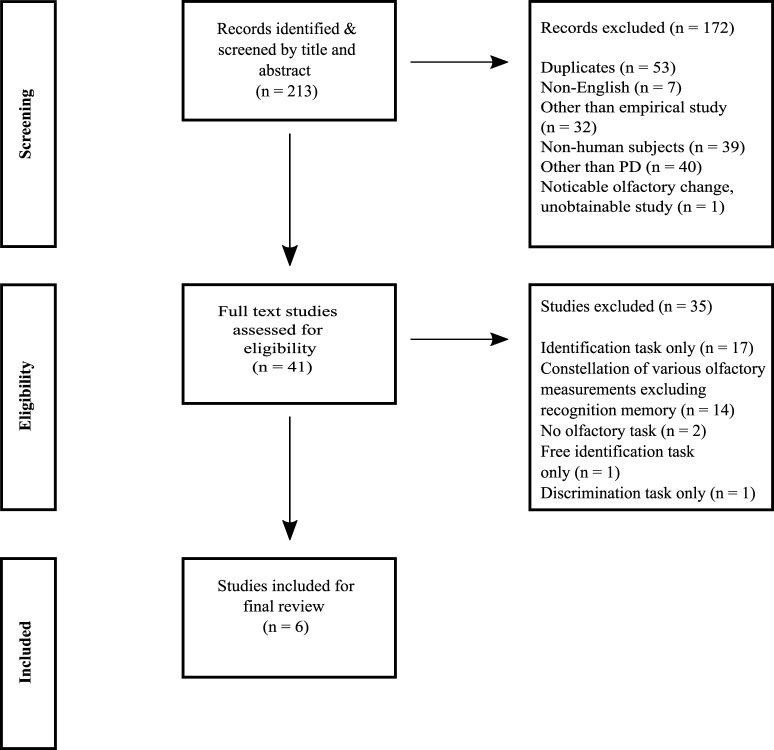
Illustration of the stepwise selection of the studies.

The selection process was performed in a stepwise manner and in accordance with the PRISMA flow diagram (see Figure 1). First, duplicates and records written in other languages than English were excluded by limitations in the search. The results correctness was confirmed by a manual comparison between the limited search and a search without any limitations. Second, further exclusion was performed based on descriptions in abstracts concerning study type, subject type, and focus on PD. When this information was unobtainable from the abstract the study's full text was screened. Third, to avoid biased inclusion of studies in the final analysis the remaining studies were full text appraised independently. The first and third author reviewed separately, which studies aimed to measure odor memory. Disagreements between the two authors were discussed with the second author (a professor in the field of olfaction and memory) and a consensus was made.

### Data Extraction and Evaluation

The extraction of data from each eligible study was derived from the PICOS-framework (see Table 1). The evaluation of the data was based on Strengthening the Reporting of Observational studies in Epidemiology (STROBE-statement). The statement contains six domains: title and abstract, introduction (rational and objectives), methods (study design, setting, participants, variables, measurement, bias, study size, and statistical methods), results (participants, descriptive data, outcome data, main results, and other analysis), discussion (key results, limitations, interpretations, generalizability), and other information (e.g., funding) (Moher et al., 2009).

**Table 1:** Summary of reviewed studies.

**References**	**Aim**	**Study design**	**Sample**	**Recognition memory task**	**Conclusion**
Boesveldt et al. (2009)	To investigate ORM in large group of patients with PD	Case-control study with analysis of variance, including odor detection scores as the main covariate	PD group (*n* = 55, 31 males and 24 females), mean age 62 years, HandY = 1–3 Control group (*n* = 50, 27 males and 23 females), mean age 59 years	Odor recognition derived from Sniffin' SticksEncoding: 8 olfactory targets were memorized, each during 3 s with 10 s intervals between the odorsRetrieval: After encoding, 8 olfactory distractors were added to the targets (in total 16 odors). Participants were asked whether an odor had been smelled before	Patients with PD performed significantly worse than controls at the odor recognition task. The difference between the groups was not significant after correction for odor detection scores
Kesslak et al. (1988)	To examine different olfactory tests for detecting AD	Case-control study with analysis of variance	PD group (*n* = 4, 3 males and 1 female), mean age 65 years, HandY = 2–5 AD group (*n* = 15, 7 males and 8 females), mean age 64 years Control group (*n* = 18, 8 males and 10 females), mean age 63 years	Odor matching with M-STEncoding: 1 olfactory target was memorized during 10 s. Retrieval: After encoding, 2 olfactory distractors were added to the target (in total 3 odors). Participants were asked to select the odor that best matched the target odor. Time interval between encoding and the correct answer in the retrieval phase was 10 s. 15 sets of the same procedure were administrated	Low correlation was found between odor matching and identification task which indicated measurement of two different olfactory functions. Few in the PD group carried out the M-ST due to anosmia assessed by the SIT (4 of 14 individuals). The patients with PD performed significantly worse in the matching task compared with the control group
Lehrner et al. (1997)	To compare olfactory threshold, odor identification ability and odor memory in AD patients and non-demented PD patients. To determine whether olfactory functions are correlated with disease progression in AD and PD	Case-control study with analysis of covariance including hit-rates, false alarm and reported disease duration as central covariates	PD group (*n* = 21, 13 males and 8 females), mean age 67 years, HandY = 1-3.5 AD group (*n* = 22, 2 males and 20 females), mean age 77 years Control group (*n* = 19, 4 males and 15 females), mean age 67 years	Odor recognition with home-made testEncoding: 10 olfactory targets were memorized, with 30 s interval between the odorsRetrieval: After 15 min, 10 olfactory distractors were added to the targets (in total 20 odors). Participants were asked to decide which odor was old alternatively new	Patients with PD performed significantly worse than controls in odor detection and identification task but not recognition
Zucco et al. (2015)	To determine if asymmetry in olfactory function could be found on odor identification and odor recognition in patients with early-stage unilateral PD	Case-control study with analysis of variance including the exposed nostril as the main covariate	PD group (*n* = 12) mean age 65 years, HandY = 1–1.5 Control group (*n* = 12) mean age 69 years. Both sexes were equally represented in the two groups	Odor matching with a combination of home-made odors and Sniffin' SticksEncoding: 1 olfactory target was memorized during 4 sRetrieval: After encoding, 3 olfactory distractors were added to the target (in total 4 odors). Participants were asked to recognize the olfactory target. Time interval between presentations of odors was 6 s. 10 sets of the same procedure with ~20 s interval were administered to each nostril	The results from the odor matching and identification task were combined to one value. The PD group's best performance (task administrated to the right nostril) was significantly worse than that of the control group
Zucco et al. (2001)	To test olfactory sensitivity in patients with early-stage unilateral PD	Case-control study with analysis of variance including the exposed nostril as the main covariate	PD group (*n* = 6) mean age 65 years, HandY = 2–2.5 Control group (*n* = 12) mean age 65 years. Both sexes were equally represented in the two groups	Odor matching with home-made testEncoding: 1 olfactory target was memorized during 4 sRetrieval: After encoding, 3 olfactory distractors were added to the target (in total 4 odors). Participants were asked to recognize the olfactory target. Time interval between presentations of odors was 6 s. 10 sets of the same procedure were administered to each nostril	Patients with PD performed significantly worse in odor matching task when the task was administered to the left nostril compared with the right nostril. No such difference was found related to the identification task. The PD group's best performance (task administered to the right nostril) was not significantly differed from that of the control group
Zucco et al. (1991)	To test the performance of PD patients on a matching and naming olfactory task	Case-control study with analysis of variance	PD group (*n* = 8, 5 males and 3 females), mean age 61 years, HandY (not reported) Control group (*n* = 16, 7 males and 9 females), mean age 80 years	Odor matching with home-made testEncoding: 1 olfactory target was memorized during 4 sRetrieval: After encoding, 3 olfactory distractors were added to the target (in total 4 odors). Participants were asked whether an odor had been smelled before. Time interval between presentations of odors was 6 s. 10 sets of the same procedure were administered	Patients with PD performed significantly better in identification task and significantly worse in the matching task compared with controls

*ORM, odor recognition memory; PD, Parkinson's disease; AD, Alzheimer's disease; MS, Multiple Sclerosis; M-ST, Match to Sample Test; SIT, Smell Identification Test; H&Y, Hoehn and Yahr Scale, describing the progression of PD*.

### Analysis and Synthesis

The differences between patients with PD and controls in performance on ORM task were measured as effect sizes for each eligible study. When information was obtainable the same comparison was performed between PD and AD patients, due to the high risk of developing dementia in PD (Hely et al., 2008). Subsequently a synthesis of outcomes from different studies was performed to allow a broader ground for conclusions. Effect sizes were calculated in accordance with *Cohen's d* (*d* > 0.50 considered to be a medium effect size) and by scripts created in R (R Core Team, 2017).

## Results

### Study Selection

The literature search generated 213 potentially relevant records. A total of 172 records were excluded due to: duplications of records, other written language than English, the articles data was not based on empirical investigation, non-human research subjects (e.g., rodents, drosophila flies and electrochemical noses), focus on other populations than individuals with PD (e.g., healthy individuals, Lewy Body Dementia, Alzheimer's disease, and Schizophrenia) and unobtainability. After exclusion, the full text of 41 studies was independently appraised concerning the studies aim to measure ORM. In total, 35 (85%) of these 41 studies did not measure odor memory. The most common measurements were odor identification (*n* = 17, 41%) or a constellation of various odor tasks such as detection, discrimination, identification, familiarity, pleasantness excluding ORM (*n* = 14, 34%). Finally, six studies (14 %) postulated to measure ORM (see Figure 1).

### Study Characteristics and Evaluation

The extraction of data from each of the six eligible studies was performed in accordance with the PICOS-framework and included description of the studies aim, design, sample, recognition memory task and conclusion (see Table 1). The extracted data focused on relevant information that assumed to shed light on the review's main questions concerning ORM in PD. Therefore, information about other tasks as odor detection, discrimination and identification, and other samples as Multiple sclerosis (MS) was not extracted although included in some studies.

The first two domains in the STROBE-statement are title and abstract, as well as introduction. All studies had relevant titles and informative abstracts. In the studies introduction, the scientific background and rationale for the investigation of numerous olfactory functions were explained. All studies incorporated an ORM task in patients with PD in relation to other study populations.

The studies method is the third evaluation domain. All studies were designed as observational case-control studies and included matched control samples. This design thought to be suitable for the studies primary objectives while secondary research questions as the relation between performance in olfactory tasks and disease duration would have benefitted more from a longitudinal design (Lehrner et al., 1997). Only two studies of the six, described the recruitment of both patients and controls (Zucco et al., 1991; Boesveldt et al., 2009). Most of the studies explained the process of the ascertainment and inclusion of cases, while information on exclusion and dropout was limited. The participants age and sex were reported in all studies and significant differences between groups were observed in two studies (Zucco et al., 1991; Lehrner et al., 1997). For example, in Lehrner et al. (1997) the AD group was significantly older and contained considerably more females compared with the PD and control group. Previous studies demonstrated that these two parameters and cultural affiliation may affect the performance in olfactory tasks (Doty and Kamath, 2014). Nevertheless, none of the studies documented the participants ethnicity. Variation between studies concerning both PD duration and stage in accordance with Hoehn and Yahr Scale (H&Y) was observed. Most of the studies focused on patients in the H&Y stage 1–3.5 while one study included also patients in H&Y stage 4–5, i.e. the advanced stages of the disease (Kesslak et al., 1988). To screen for dementia, most studies used short cognitive screening tools as Mini-Mental State Examination (MMSE). Two studies did not report a cognitive screening of PD patients but postulated that the patients did not show any signs of dementia (Zucco et al., 1991, 2001). Thus, studies had varied inclusion criteria concerning cognitive performance while no study reported inclusion of patients with PDD.

In general, the recruited PD samples in the eligible study population were small ranging between four to 55 patients. Furthermore, one study partly incorporated the same participants from a previous study that also had been included in the reviewed study population (Zucco et al., 2015). Additionally, few studies (*n* = 2) included small AD samples (Kesslak et al., 1988; Lehrner et al., 1997). The instruments and procedures to measure ORM were documented thoroughly in all studies. Measurements were varied and divided into two main categories: odor matching (*n* = 4) and recognition task (*n* = 2). Three of the four matching tasks were administrated in the same manner while the two recognition tasks were different, especially regarding to the retention interval (see Table 1). Multiple statistical tools were used to treat the data while analysis of variance that included possible influential covariates as age, sex, disease duration etc. was the main statistical analysis in all studies.

The last three domains in the statement are results, discussion, and other information. All studies described the results concerning the odor memory recognition tasks. One of six studies did not report separately the odor matching scores but incorporate them with performance on odor identification task to one olfactory quota (Zucco et al., 2015). Discussions included key results and interpretations, but problematization of studies generalizability and limitations was scarce. Some studies commented the limited generalizability of results due to the small sample sizes (Zucco et al., 2001). None of the studies reflected on the problem with generalizability regarding varied ORM tasks and the need for standardized measurement. Only two of the six studies reported information about their funding of the research (Kesslak et al., 1988; Boesveldt et al., 2009).

### Analysis and Synthesis

The effect sizes between different samples; PD, AD, and controls were calculated for each eligible study in accordance with *Cohen's d*. Zucco et al. (2015) was excluded from the statistical analysis due to partially inclusion of the same participants in another eligible study (Zucco et al., 2001). Furthermore, the scores in the odor matching task were not reported separately but were merged with performance in odor identification task, to one olfactory quota (Zucco et al., 2015).

Synthesis of the two studies including the odor recognition task resulted in PD sample (*n* = 76) and control sample (*n* = 69) (Lehrner et al., 1997; Boesveldt et al., 2009). The PD group performed significantly lower than controls and the difference between the groups was medium in accordance with Cohen's *d* (0.60). The same synthesis could not be performed in relation to the AD sample since only one study of the two included such a sample. Nevertheless, based on this one study the patients with PD (*n* = 76) performed better than the AD (*n* = 22) patients in the odor recognition task (Lehrner et al., 1997). The difference between the two groups was slightly higher (*d* = 0.68).

In relation to the odor matching task three studies were merged which resulted in PD sample (*n* = 18) and controls (*n* = 46) (Kesslak et al., 1988; Zucco et al., 1991, 2001). PD patients performed significantly lower than controls and the difference between the groups was noticeably large (*d* = 1.39). Only one study of the three, included AD patients and therefore a synthesis concerning this group was not possible. Based on that one study, no significant difference between PD and AD patients in performance on odor matching task was observed (*d* = 0.48) (Kesslak et al., 1988).

## Discussion

The literature on ORM in PD was limited and only six eligible studies on the subject were found. The sample sizes were generally small. Differences within and between studies concerning inclusion criteria, sample size, sex ratio, age, disease stage, disease duration, and performance in cognitive tests indicated heterogenous data. This partially explained a previous meta-analysis result demonstrated that the highest inconsistency in studies was related to AD and PD patients performance in ORM tasks (*I*^2^ = 87.7%) (Rahayel et al., 2012). Additionally, variation between reviewed studies was discovered regarding the design of the ORM task. Two main task designs were identified and categorized in this review as odor matching and odor recognition task. The odor matching task resembled measurements of odor discrimination and therefore theoretically is more related to working memory. Conversely, the design of the odor recognition task included more olfactory targets per set and longer time intervals of encoding and retention suggests greater involvement of episodic memory (Larsson, 2002; Hedner et al., 2010; Croy et al., 2015).

In contrast to Rahayel et al. (2012), where odor matching and recognition were considered to measure the same olfactory function (i.e., ORM), this review investigated the tasks separately, due to discrepancies in design and hence hypothetically measuring two different olfactory functions. In line with this approach, the review's synthesis revealed variation in effect sizes. PD patients showed worse performance than controls in the odor recognition task but especially in the odor matching task. Hence, tasks targeting odor matching and odor discrimination may therefore serve as markers for identifying prodromal PD allowing early treatment. However, a moderate effect size indicating that PD patients performed better than patients with AD was observed only in relation to the odor recognition task.

These findings are in accordance with the theory that odor recognition task, which requires greater involvement of odor episodic memory, is more sensitive to signs of dementia. Consequently, the systematic review generates the hypothesis that odor recognition task may have the potential to identify patients with *de novo* PDD and therefore improve the choice of treatment, especially in the advanced phases of the disease. A careful differentiation between patients with PD and those with PDD is of importance since some treatments as deep brain stimulation in subthalamic nucleus (DBS-STN) have been associated with decline of cognitive function (Witt et al., 2013). Still, it is important to highlight some limitations of the present review. First, given that few studies have addressed ORM in PD and that available evidence show large heterogeneity, any generalization should be drawn with caution. Moreover, impaired olfactory sensitivity has been associated to ORM in PD and therefore the performance in ORM may be highly depended on the ability to detect odors (Boesveldt et al., 2009). Nevertheless, these two tasks demonstrate differences regarding structural and behavioral data (Savic et al., 2000). Future work should adopt longitudinal prospective designs to study ORM in different PD stages, PDD, and AD.

## Author Contributions

TE conceptualized and implemented the systematic review. ND and ML evaluated studies eligibility and commented the article. All authors approved the final version of the paper and agreed to be accountable for all aspects of the work.

## Conflict of Interest

The authors declare that the research was conducted in the absence of any commercial or financial relationships that could be construed as a potential conflict of interest.
